# Case Report: Metagenomic next-generation sequencing diagnosed a rare case of sternal tuberculosis mimicking a malignant tumour

**DOI:** 10.3389/fmed.2025.1708834

**Published:** 2026-01-14

**Authors:** Jiawei Huang, Cong Lan, Yunjie Liang, Huilong Chen, Hanping Liang, Haiquan He, Siyao Che, Ying Chen

**Affiliations:** 1 Department of Thoracic Surgery, Affiliated Gaozhou People’s Hospital, Guangdong Medical University, Maoming, Guangdong, China; 2 Guangdong Medical University, Zhanjiang, Guangdong, China; 3 Affiliated Gaozhou People’s Hospital, Guangdong Medical University, Maoming, Guangdong, China

**Keywords:** sternal tuberculosis, metagenomic next-generation sequencing, tuberculosis, diagnosis, treatment

## Abstract

This is a case report of a 17-year-old female patient who presented with a painless, palpable swelling on the anterior chest wall. Imaging studies revealed osteolytic lesions involving the manubrium and adjacent ribs, along with multiple enlarged lymph nodes, raising a high suspicion of malignant tumour with metastasis. An ultrasound-guided needle biopsy revealed the pathological finding of “granulomatous inflammation.” Multidisciplinary consultation and clinical indicators, including a strongly positive purified protein derivative (PPD) test and markedly elevated erythrocyte sedimentation rate, were taken to indicate a potential diagnosis of tuberculosis. Consequently, subsequent metagenomic next-generation sequencing (mNGS) of the biopsy specimen identified nucleic acid sequences belonging to the *Mycobacterium tuberculosis* complex, thereby confirming the rare diagnosis of sternal tuberculosis. Following the administration of standardised anti-tuberculosis therapy, a substantial reduction in the size of the lesion was observed, thereby validating the accuracy of the diagnosis. This case underscores the importance of considering extrapulmonary tuberculosis in the differential diagnosis of bone-destructive lesions and demonstrates the critical value of mNGS technology in confirming challenging infectious diseases.

## Introduction

Thoracic wall tuberculosis is an extremely rare form of extrapulmonary tuberculosis. Due to its clinical manifestations and radiographic features often resembling those of metastatic malignant tumours, it frequently results in misdiagnosis and delayed treatment. This study reports a case of sternal tuberculosis that initially presented as a thoracic wall mass with bone destruction and was ultimately confirmed by metagenomic next-generation sequencing (mNGS).

## Case

**Figure 1:**
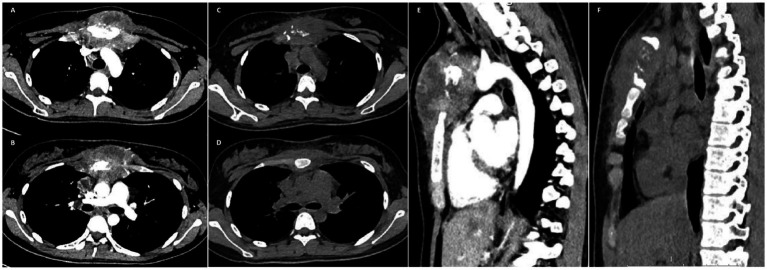
**(A,B)** Initial chest computed tomography (CT) revealed osteolytic destruction of the manubrium with associated soft tissue swelling. **(C,D)** Follow-up chest CT performed approximately 4 weeks after initiation of anti-tuberculosis therapy revealed a reduction in soft tissue swelling surrounding the manubrium. **(E)** Sagittal chest CT at baseline shows the sternal lesion. **(F)** Follow-up sagittal chest CT shows significant improvement of the sternal lesion compared to the baseline.

A 17-year-old female patient presented to Gaozhou People’s Hospital on 20 July 2025 with a chief complaint of a painless, protruding mass on the anterior chest wall that had been noticed 3 months earlier. She reported no systemic symptoms such as fever, night sweats, cough, sputum production, or weight loss. Physical examination revealed a firm, non-tender mass with limited mobility, which was palpable at the manubrium of the sternum. The overlying skin showed no erythema or ulceration. Chest computed tomography (CT) revealed osteolytic destruction of the manubrium with associated localised soft tissue swelling measuring approximately 94 mm × 52 mm, exhibiting ill-defined borders and heterogeneous density. Localised destruction was also noted in the adjacent left first and third anterior ribs. Multiple enlarged lymph nodes were identified in the right supraclavicular fossa, mediastinum, and hilar regions (Figure 1). As shown in Table 1, laboratory findings included mildly elevated white blood cell and platelet counts. Notably, multiple tumour markers were within normal ranges, and human immunodeficiency virus (HIV) testing was negative.

**Table 1:** Partial inspection results and reference range.

Variable	Result	Reference range
AFP	<0.91	0–7.00 ng/mL
CEA	0.88	0–4.7 ng/mL
CA19-9	4.28	0–39.00 U/mL
CA72-4	1.49	0–6.90 U/mL
CA15-3	25.7	0–34.50 U/mL
CA125	16.1	0–35.00 U/mL
SCC	0.98	0–2.70 ng/mL
β-HCG	<0.20	≤4.9 mIU/mL (non-pregnancy)
WBC	10.32 ↑	4.00–10.00 × 10^9^/L
RBC	4.42	3.68–5.13 × 10^12^/L
HGB	113	113–151 g/L
PLT	638 ↑	100–400 × 10^9^/L
ALT	9	7–40 U/L
AST	19	13–35 U/L
ALB	42.9	35.0–55.0 g/L
Total bilirubin	6.6	5.10–17.10 μmol/L
PCT	0.03	0–0.050 ng/mL
HIV testing	Negative	/
ESR	102	0–20.00 mm/h
Sputum culture	Negative	/
PPD test	Strongly positive	/
T-spot	Positive	/
mNGS	*Mycobacterium tuberculosis* complex	/

AFP, alpha-fetoprotein; ALB, albumin; ALT, alanine aminotransferase; AST, aspartate aminotransferase; CA125, carbohydrate antigen 125; CA15-3, carbohydrate antigen 15-3; CA19-9, carbohydrate antigen 19-9; CA72-4, carbohydrate antigen 72-4; CEA, carcinoembryonic antigen; ESR, erythrocyte sedimentation rate; HGB, haemoglobin; HIV, human immunodeficiency virus; mNGS, metagenomic next-generation sequencing; PCT, procalcitonin; PLT, platelet count; PPD, purified protein derivative; RBC, red blood cell; SCC, squamous cell carcinoma antigen; WBC, white blood cell; β-HCG, beta human chorionic gonadotropin.

Based on the imaging results, the preliminary clinical diagnosis was “malignant mediastinal tumour invading the sternum and ribs with lymph node metastasis.” Consequently, an ultrasound-guided biopsy of the mediastinal mass was performed on 21 July 2025. The postoperative pathology report indicated “granulomatous inflammation,” with no pathogens detected using special stains (PAS, hexamine silver, and acid-fast staining).

In view of the marked discrepancies between pathological findings and clinical imaging assessments, a multidisciplinary team (MDT) consultation was convened at our hospital. Following a period of deliberation, it was determined that the patient, a young female exhibiting normal tumour markers, exhibited no conventional symptoms associated with tuberculous toxaemia. On further questioning, both the patient and her family denied any history of contact with individuals with pulmonary tuberculosis or travel to high-prevalence regions. Nevertheless, high vigilance for infectious diseases, particularly extrapulmonary tuberculosis, was warranted. Subsequent tuberculin skin testing (purified protein derivative, PPD test) yielded a strongly positive reaction (induration of 25 mm), T-SPOT testing was positive, and the erythrocyte sedimentation rate (ESR) was markedly elevated (102 mm/h). However, multiple sputum smears yielded negative results for acid-fast bacilli and serum tuberculosis antibody testing (Table 1).

Following a second MDT discussion, the diagnosis was strongly suggestive of “tuberculosis of the sternum and ribs,” despite the absence of definitive pathogenetic evidence. To confirm the diagnosis, mNGS was performed on the previously obtained needle biopsy tissue sample. The testing, conducted by Guangzhou Huayin Medical Laboratory Center using the MGI200 platform, detected nucleic acid sequences specific to the *Mycobacterium tuberculosis* complex.

The final diagnosis was “tuberculosis of the sternum and ribs.” The patient was referred to a specialist hospital for chronic diseases to receive the standard four-drug anti-tuberculosis regimen (isoniazid, rifampicin, pyrazinamide, and ethambutol). Approximately 4 weeks into the treatment regimen (2 September 2025), the patient underwent a follow-up examination. A thorough clinical evaluation was conducted, which revealed a significant decrease in the dimensions of the chest wall mass when compared to prior assessments. Follow-up chest computed tomography (CT) revealed a significant decrease in the extent of the original sternal body destruction area (approximately 62 mm × 42 mm) and reduced surrounding soft tissue oedema (Figure 1), confirming therapeutic efficacy.

## Discussion

Tuberculosis (TB) remains a significant global health challenge and is often termed “a great imitator” due to its diverse and atypical clinical manifestations (1, 2). This case exemplifies this characteristic, as the presenting imaging features—osteolytic destruction of the sternum and ribs accompanied by multiple enlarged lymph nodes—closely mimicked those of primary malignancies such as osteosarcoma or lymphoma, as well as metastatic disease, leading to initial diagnostic uncertainty.

In tuberculosis, beyond the common pulmonary presentations, extrapulmonary disease can involve multiple organ systems. The reported site distribution is as follows: lymphatic system (~59.1%), abdominal region (~15.9%), osteoarticular and spinal structures (~13.6%), urogenital system (~4.5%), pleura (~4.5%), and skin (~2.3%) (3). Within the skeletal system, tuberculosis most commonly affects the spine, followed by the hip, knee, and ankle joints (4).

Thoracic wall tuberculosis is an uncommon form of extrapulmonary tuberculosis, most commonly affecting the sternum and ribs. The presence of symptoms such as cough, sputum production, night sweats, and afternoon fever may be indicative of tuberculosis. As reported in extant literature, fever and localised pain are prevalent initial symptoms of sternal tuberculosis (5–7). Sinus tract formation is also frequently observed (7, 8). Nonetheless, painless swelling as the primary symptom is an uncommon occurrence and has been sporadically documented (1, 9, 10). It has been documented that some patients have been identified as concurrently infected with HIV (4). Typically, computed tomography (CT) and magnetic resonance imaging (MRI) scans show osteolytic lesions in the affected sternum, accompanied by swelling of the adjacent soft tissues (11).

The definitive diagnosis of extrapulmonary tuberculosis has traditionally relied upon bacteriological or histological identification of *Mycobacterium tuberculosis* from lesion specimens, with culture and acid-fast staining considered the gold standard, typically achieved through needle aspiration or surgical biopsy (8). However, as demonstrated in this case, these conventional methods often exhibit low sensitivity for extrapulmonary mycobacterial disease. Furthermore, while traditional immunological methods such as the PPD test have been widely used, their results can be influenced by prior Bacille Calmette–Guérin (BCG) vaccination or exposure to non-tuberculous mycobacteria, potentially leading to false-positive results. Conversely, false-negative results may occur in some patients with active tuberculosis or those who are immunocompromised. Consequently, the PPD test is primarily used for epidemiological screening rather than as a standalone diagnostic tool for confirming active tuberculosis. The patient’s diagnosis was ultimately confirmed by mNGS, which detected nucleic acid sequences of the *Mycobacterium tuberculosis* complex, rather than conventional microbiology (negative). This advanced molecular technique is increasingly recognised by guidelines, including those of the World Health Organization (WHO), as a vital diagnostic tool for challenging cases (12). The subsequent marked radiological and clinical improvement observed after the initiation of standard four-drug anti-tuberculosis therapy further corroborated this diagnosis, underscoring that the combination of modern molecular evidence and a positive therapeutic response can serve as a reliable basis for confirmation, even in the absence of classical bacteriological evidence.

There is no universally accepted consensus on the treatment of sternal tuberculosis. Notwithstanding, the prognosis is generally favourable. The primary treatment regimen consists of first-line multi-drug anti-tuberculosis chemotherapy, with a standard course of 9–12 months in the absence of microbial drug resistance (1, 13). Research indicates that early and adequate pharmacological intervention can typically obviate the need for surgery (7, 14). When surgical intervention is required (e.g., for extensive abscess formation, significant sequestra, or failed medical therapy), it typically comprises thorough drainage, complete debridement, and sternotomy. Subsequent studies have shown that patients receiving standardised treatment (encompassing first- and second-line therapy and surgical debridement) ultimately achieve complete resolution without recurrence (7).

## Conclusion

Sternal tuberculosis is a rare great imitator that can perfectly mimic malignancy. This case highlights that mNGS is pivotal for definitive diagnosis when conventional methods are negative. TB should be considered in destructive bone lesions even without typical symptoms, as timely diagnosis prevents unnecessary interventions and ensures effective treatment.

## Data Availability

The original contributions presented in the study are included in the article/supplementary material, further inquiries can be directed to the corresponding authors.
